# RESEARCH NOTE: Effect of inhibition of bone morphogenetic protein signaling pathway by dorsomorphin on the establishment of embryo polarity

**DOI:** 10.1016/j.psj.2026.106644

**Published:** 2026-02-16

**Authors:** Hyung Chul Lee

**Affiliations:** a School of Biological Sciences and Technology, College of Natural Sciences, Chonnam National University, 77 Yongbong-ro, Gwangju 61186, Korea; b Institute of Plant Biomaterials and Biomedical Science, Chonnam National University, 77 Yongbong-ro, Gwangju 61186, Korea

**Keywords:** Chick embryo, BMP, Primitive streak, Dorsomorphin, Embryo polarity

## Abstract

Bone morphogenetic proteins (BMPs) play a crucial and conserved role in establishing embryo polarity across the animal kingdom. However, it is not known how different levels of BMP activity affect embryo polarization in amniote species. To address this question, here we treat dorsomorphin, a selective inhibitor of the BMP signaling, at various concentrations, on early chick embryos and observe the effect on primitive streak formation. In our results, dorsomorphin affects primitive streak formation in a dose-dependent manner, gradually inhibiting streak morphology from the anterior to the posterior direction. Additionally, at higher concentrations, dorsomorphin induces enhanced morphological change in isolated anterior cut-half embryos. Taken together, our results suggest that BMP inhibition by dorsomorphin not only inhibits the formation of the anterior primitive streak but also, at higher concentrations, indirectly inhibits the posterior region, possibly by the ectopic recruitment of cells in the non-posterior areas.

## Introduction

During gastrulation, extensive cellular migration is accompanied by the differentiation of cells into mesoderm and endoderm, forming the three germ layers. In amniote species, gastrulation begins with the formation of the primitive streak, a midline structure that elongates gradually from a future posterior to an anterior direction. As the formation of the primitive streak determines the anterior-posterior and the left-right axis, its proper development is crucial for the body plan. Bone morphogenetic protein (BMP) signaling, acting with WNT, is conserved in early embryo patterning (Petersen and Reddien, 2009). Along the anterior-posterior axis, BMP shows a gradient in its expression and activity. In chick embryos, BMP activity forms a graded pattern along the future anterior–posterior axis, and perturbing this gradient disrupts normal axial development (Streit et al., 1998).

In the chick embryo, before the formation of the primitive streak, when the embryo still exhibits radial symmetry, *BMP* and its downstream targets (e.g., *GATA2*) are expressed in the marginal zone in an anterior–posterior gradient (Bertocchini and Stern, 2012). Recent work suggests that this long-range pattern can arise from intercellular propagation of calcium firing activity through gap junctions, which modulates BMP activity via NF-κB and NFAT (Lee et al., 2024). The calcium firing activity regulates BMP activity through the transcription factors NF-κB and NFAT, generating the BMP gradient along the anterior-posterior axis. In the cells at the site of the lowest BMP activity out of the gradient, *cVG1* is expressed, and the primitive streak starts to form in the inner area pellucida. It was suggested that epiblast cells in the marginal zone sense changes in cVG1/BMP signal levels relative to their neighboring cells to assess their position in forming a primitive streak (Lee et al., 2022). However, how different degrees of BMP inhibition translate into distinct primitive streak outcomes remains unclear.

Dorsomorphin is a selective inhibitor of BMP signaling through type I receptors, including ALK2, ALK3, and ALK6 (Yu et al., 2008). In studies using the chick embryo, it has been demonstrated that dorsomorphin can effectively inhibit BMP signaling, inducing a single or multiple ectopic primitive streaks when applied locally or globally, respectively, to non-posterior regions (Lee et al., 2024). Similar ectopic streaks can be induced by local application of chordin (Streit et al., 1998). In this study, we administer graded doses of dorsomorphin to early chick embryos to test dose-dependent effects on primitive streak formation. We find that dorsomorphin has a dose-dependent effect on primitive streak formation and embryo development.

## Materials and methods

### Embryo manipulation and whole-mount in situ hybridisation

Embryos at EGK stage X-XI from fertilized White Leghorn (*Gallus gallus domesticus*) hens’ eggs (Henry Stewart, UK) were harvested. The embryos were cultured *ex-ovo* (Stern and Ireland, 1981) for 21 hours before fixation. To make anterior half-embryos, embryos at EGK stage X-XI were cut in half using a syringe needle before culture. Whole-mount *in situ* hybridization was performed as previously described (Stern, 1998). The probes used were: *cVG1* (*GDF3*) (Shah et al., 1997) and *Brachyury* (*cBRA, TBXT*) (Kispert et al., 1995). Stained embryos were imaged with an Olympus SZH10 stereomicroscope with a QImaging Retiga 2000R camera.

### Dorsomorphin treatment and scoring embryo morphology

For dorsomorphin (Tocris, 3093) treatment to the whole embryo, it was diluted first in phosphate-buffered saline (1:10 v:v) and then in albumen (9:10 v:v), which was used for ex ovo culture. Dimethyl sulfoxide (DMSO), a dilution agent for dorsomorphin, was used in the control groups at a final concentration of 0.2%, which is the highest concentration used in the treatment groups (e.g., 20 μM of dorsomorphin).

In this study, embryos treated with various concentrations of dorsomorphin exhibited three distinct morphologies, based on the formation of the primitive streak, which were clearly distinguishable from each other: type 1, a normal primitive streak with a linear shape; type 2, an anteriorly deformed primitive streak while maintaining a linear shape on the posterior side; and type 3, a ring structure without any streak morphology.

### Graphs and statistical analysis

We used GraphPad Prism version 6.01 for drawing all graphs and statistical analysis. An unpaired two-sided Student’s t-test and Chi-square test were used.

## Results and discussion

### Effect of BMP inhibition on primitive streak formation in a dose-dependent manner

**Fig. 1:**
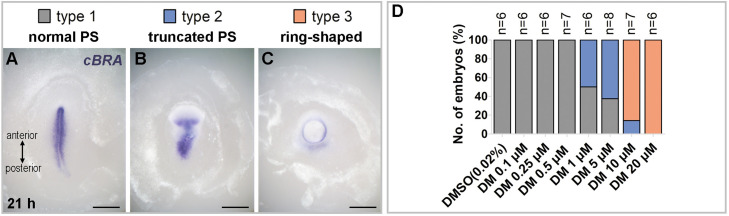
**Dose-dependent effect of dorsomorphin on primitive streak formation.** Chick embryos at EGK stage X-XI are cultured *ex-ovo* for 21 hours, with administration of different concentrations of dorsomorphin. (A-C) Representative images showing three types of morphology of the primitive streak: normal (A), truncated (B), and ring-shaped (C). *cBRA* expression marks primitive streak cells. Orientation of embryos is indicated in (A). (D) Numerical data of the morphological types at various concentrations of dorsomorphin (DM). n for each condition: 6, 6, 6, 7, 6, 8, 7, and 6, in order of increasing the concentration of dorsomorphin (0, 0.1, 0.25, 0.5, 1, 5, 10, and 20 μM). Chi-square test: *p* = 1.56e-08. Scale bars: 1 mm.

To investigate how different BMP activities affect primitive streak formation in early chick embryos, various doses of dorsomorphin were administered to the whole embryos (Fig. 1). Formation and morphology of the primitive streak were examined by in situ hybridization with a primitive-streak-marker, *BRACHYURY* (*cBRA*). Throughout the various concentrations, the resulting embryo morphologies can be classified into three types: normal, truncated, and ring-shaped primitive streak (Fig. 1A-C). In type 2, the anterior part of the primitive streak showed shortened and truncated morphology, while its posterior part was normal (Fig. 1B). In type 3, the streak morphology was lost, and ring-shaped expression of *cBRA* was observed.

**Fig. 2:**
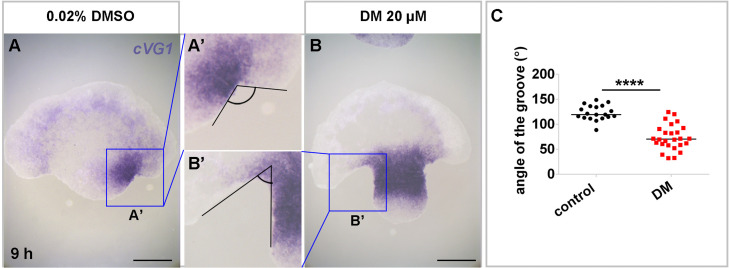
**Enhanced morphological change in isolated anterior cut-half embryos by dorsomorphin treatment**. Isolated anterior cut-half embryos are cultured *ex-ovo* for 9 hours with administration of 0.02% DMSO (control) or 20 μM dorsomorphin. (A) 0.02% DMSO. (B) 20 μM dorsomorphin (DM). A’ and B’ are magnified views of the boxed region in (A) and (B), respectively. *cVG1* expression marks the primitive-streak forming region. (C) Comparison of the angle of the groove between the control and DM treatment. Dots represent each data. *n* = 17 and 26 in the control and DM groups, respectively. Unpaired Student’s t-test (two-sided): ****p < 0.0001. Scale bars: 1 mm.

The effect of dorsomorphin is dose-dependent (Fig. 2). The control solution (0.2% DMSO) did not affect the embryos. At 0∼0.5 μM, embryos were normal (type 1) with proper primitive streak formation. Between 1∼5 μM, more than 50% of embryos showed anteriorly truncated morphology (type 2). At 10∼20 μM of dorsomorphin, most of the embryos showed ring-shaped morphology. Taken together, it appears that dorsomorphin affects primitive streak formation, progressing from the anterior to the posterior region, as the concentration of dorsomorphin increases.

### Potent BMP inhibition causes global cellular movement that inhibits the formation of the morphology of the primitive streak

The dose-dependent effect of dorsomorphin on the embryo seems to be explained by the existing BMP gradient in the early embryo, which is higher in the anterior and gradually decreases towards the posterior (Lee et al., 2022). In this regard, the treatment of dorsomorphin to the whole embryo would affect the anterior side, where BMP, a dorsomorphin target, is in action, but not in the anterior, where BMP is low and less active. Indeed, the intermediate concentration of dorsomorphin (1∼5 μM) affects the anterior region of the primitive streak as in the type 2 (Fig. 1). However, this does not explain the effect on the posterior side when the higher concentration (10∼20 μM) of dorsomorphin was treated, because posterior side has very low BMP expression and activity (Lee et al., 2024) in which dorsomorphin is expected to have no effect in the absence of its target. Then, what causes this abnormal morphology on the posterior side? One possibility is that the high concentration of dorsomorphin might have a BMP-independent effect or cytotoxicity. Dorsomorphin is also known as an AMPK inhibitor. However, a previous study showed BMP receptor inhibitor LDN also exhibited a similar effect (Chuai et al., 2023), suggesting that type 3 morphology with a high concentration of dorsomorphin is BMP-dependent as well (Lee et al., 2024). Additionally, cytotoxicity does not appear to be a cause, as prominent cellular death, which would result in holes in the blastoderm during embryo expansion, has not been observed in this study. Also, it was confirmed that 20 μM of dorsomorphin (the highest concentration in this study), along with the same administration method, has no cytotoxicity (Lee et al., 2024). Furthermore, isolated cut embryos treated with the same concentration showed no morphological defects. Nevertheless, we cannot fully exclude contributions from off‑target effects of dorsomorphin.

Another possible cause is that 10∼20 of dorsomorphin has a more substantial effect in the non-posterior region of the embryo, which causes a secondary impact on the posterior side by cellular activity at the tissue level. During gastrulation, a massive cellular movement, known as the polonaise movement, occurs in the epiblast to form a primitive streak, involving the recruitment of lateral and anterior cells towards the posterior side (Voiculescu et al., 2014). Therefore, if there is ectopic recruitment of cells in the non-posterior region, original primitive streak formation should be defective due to insufficiency of cells. Indeed, BMP inhibition by dorsomorphin treatment leads to the ectopic formation of the primitive streak, accompanied by ectopic *cBRA* expression, which would recruit ectopically epiblast cells. To confirm this possibility, 20 μM of dorsomorphin was applied to isolated anterior half cut embryos, which spontaneously generate an ectopic primitive streak at either the left or right side (Fig. 2) (Lee et al., 2024). After 9 hours of culture, the embryonic pieces were examined for *cVG1* expression, which marks the site of the primitive streak formation (Shah et al., 1997). The control embryos (0.02% DMSO) showed *cVG1* expression with a minor groove (indentation of the epithelial cell sheet) on either side, indicating formation of the primitive streak with possible cellular movement (Fig. 2A). With this localized *cVG1* expression, the isolated anterior cut halves can generate a proper anterior-to-posterior axis (Lee et al., 2024). On the other hand, dorsomorphin-treated embryos exhibited *cVG1* expression throughout the marginal zone (Fig. 2B), as observed in the whole embryo treated with dorsomorphin (Lee et al., 2024). Additionally, the global expression of *cVG1* along the marginal zone was accompanied by an extensive groove on both sides, making an improper embryonic axis; only the posterior region, rather than the antero-posterior axis (Fig. 2B and C). Taken together, these results suggest that ring-shaped primitive streak formation following BMP inhibition may arise from ectopic induction of streak-like behavior outside the posterior region (e.g., expanded *cVG1* and associated ingression/mesendodermal movements). We propose that these ectopic movements compete with and distort the normal posteriorly initiated polonaise movement, thereby misdirecting cell flows and ultimately leading to posterior patterning defects.

## CRediT authorship contribution statement

**Hyung Chul Lee:** Writing – review & editing, Writing – original draft, Visualization, Validation, Supervision, Methodology, Investigation, Funding acquisition, Data curation, Conceptualization.

## Disclosures

The authors declare that they have no known competing financial interests or personal relationships that could have appeared to influence the work reported in this paper.
